# Poem Taps

**Published:** 1919-03

**Authors:** John T. Troth


### TEXAS MEDICAL JOURNAL

DR. F. E. DANIEL, Founder.

Established July, 1885

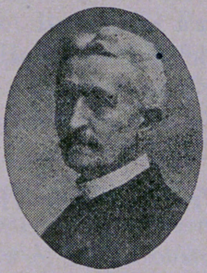

MRS. F. E. DANIEL, Publisher and Managing Editor.

VOL. XXXIV No. 9
AUSTIN,
MARCH, 1919.

PUBLISHED MONTHLY.
Subscription:
$1.50

The publisher is not responsible for views of contributors.

ASSISTANT EDITORS:

DR. JOHN S. TURNER, Dallas, Texas. DR. JOE BECTON, Greenville, Texas.
DR. E. B. PARSONS, Palestine, Texas. DR. M. F. BLEDSOE, Port Arthur.
DR. A. P. HOWARD, Houston, Texas. DR. J. H. FLORENCE, Tulsa, Oklahoma.
DR. J. H. REUSS, Cuero, Texas.

### EDITORIAL.

### Taps!

BY JOHN T. TROTH

Eddie, an' Jim, an' Squint-eye Joe,
Barefooted, freckled, an' tanned,
Lay on their backs, with their moth-eaten pup,
In the warm September glow;
An' told what they'd be when they growed up—
Eddie, an', Jim, an' Joe!

Eddie, an' Jim, an' Squint-eye Joe
Were bound to be richer'n kings!
Eddie's ambition a judge's wig—
Jim would explorin' go,
An' Joe'd be a actor, when *he* got big—
Eddie, an' Jim, an' Joe!

Eddie, an' Jim, an' Squint-eye Joe
Lie on the shell-torn earth!
Jim drags Joe to a crater's brink,
Where Eddie, dyin' below,
Beckons, an' gives 'em his last drop to drink—
Eddie, an' Jim, an' Joe!

Eddie, an' Jim, an' Squint-eye Joe
Lie on their backs, asleep:
Their Great Adventure has come, and passed,
And crosses three, in a row,
Tell that they're richer than kings, at last—
Eddie, an' Jim, an' Joe!

—Everybody's Magazine.

